# Platelet phosphatidylserine exposure and microparticle production as health bioindicators in marine mammals

**DOI:** 10.3389/fvets.2024.1393977

**Published:** 2024-05-10

**Authors:** Mar Felipo-Benavent, Mónica Valls, Maria Céu Monteiro, Beatriz Jávega, Daniel García-Párraga, Consuelo Rubio-Guerri, Alicia Martínez-Romero, José-Enrique O’Connor

**Affiliations:** ^1^Laboratory of Cytomics, Joint Research Unit CIPF-UVEG, Department of Biochemistry and Molecular Biology, University of Valencia, Valencia, Spain; ^2^Department of Biomedical Sciences, Faculty of Health Sciences, Universidad CEU Cardenal Herrera, CEU Universities, Valencia, Spain; ^3^Veterinary Services, Oceanogràfic, Ciudad de las Artes y las Ciencias, Valencia, Spain; ^4^1H-TOXRUN—One Health Toxicology Research Unit, University Institute of Health Sciences (IUCS), CESPU, CRL, Gandra, Portugal; ^5^Research Department, Fundación Oceanogràfic de la Comunitat Valenciana, Valencia, Spain; ^6^Department of Pharmacy, Faculty of Health Sciences, Universidad CEU Cardenal Herrera, CEU Universities, Valencia, Spain; ^7^Cytomics Technological Service, Príncipe Felipe Research Center, Valencia, Spain

**Keywords:** hemostasis, calcium ionophore, Annexin V, bottlenose dolphin, sea lion, beluga whale, walrus, CD41

## Abstract

In human medicine, various pathologies, including decompression sickness, thrombocytopenia, and rheumatoid arthritis, have been linked to changes in cellular microparticles (MP) formation, particularly platelet microparticles (PMP). Similar disorders in marine mammals might be attributed to anthropogenic threats or illnesses, potentially impacting blood PMP levels. Thus, detecting platelet phosphatidylserine (PS) exposure and PMP formation could serve as a crucial diagnostic and monitoring approach for these conditions in marine mammals. Our group has developed a methodology to assess real-time PS exposure and PMP formation specifically tailored for marine mammals. This method, pioneered in species such as bottlenose dolphins, beluga whales, walruses, and California sea lions, represents a novel approach with significant implications for both clinical assessment and further research into platelet function in these animals. The adapted methodology for evaluating PS exposure and PMP formation in marine mammals has yielded promising results. By applying this approach, we have observed significant correlations between alterations in PMP levels and specific pathologies or environmental factors. These findings underscore the potential of platelet function assessment as a diagnostic and monitoring tool in marine mammal health. The successful adaptation and application of this methodology in marine mammals highlight its utility for understanding and managing health concerns in these animals.

## 1 Introduction

Microparticles (MP) are vesicles derived from cell membranes with diameters ranging between 0.1 μm and 1 μm, having important functions in cell communication (1). Among MP, platelet microparticles (PMP) are the most abundant in the bloodstream of healthy animals (1–3), constituting 70–90% of MP and presenting a wide variety of preanalytic variables and analytic variables, resulting in a wide range of PMP values in platelet-free plasma (PFP) of healthy subjects (100–4,000 PMPs μL^−1^). These data indicate that standardization of PMP enumeration by flow cytometry is feasible but is dependent on intrinsic characteristics of the flow cytometer and the calibration strategy (4). PMP, which contain CD41, are physiologically produced during the final phase of platelet activation. Following intraplatelet Ca^2+^ mobilization, shape alteration, aggregation, and granule secretion, platelets manifest procoagulant activity by exposing phosphatidylserine (PS) from the inner to the outer face of the platelet membrane (5). Subsequently, small membrane fragments are released as PMP (5), playing crucial roles in cell communication, transporting bioactive molecules, and signaling various processes associated with hemostasis and thrombosis.

Moreover, various pathologies are linked to alterations in PMP production, such as decompression sickness (1), thrombocytopenia, rheumatoid arthritis (6), cancer (3, 7, 8), arterial thrombosis (9) atherosclerosis (10), immune thrombocytopenic purpura, or even malaria infection (3). Alterations in blood PMP levels can be associated with different risks. On the one hand, elevated PMP levels can lead to platelet deposition and thrombus formation (3). On the contrary, reduced levels are associated with a propensity for bleeding, as occurs in Castaman’s defect or Scott’s syndrome (11).

For this reason, monitoring blood PMP production serves as a valuable diagnostic and monitoring tool for several disorders, including cancer (12). Despite some of these diseases are not necessarily documented in marine mammals, this is likely due to limited information available compared to other mammals, stemming from the challenges of accessing these creatures in the sea rather than a lack of interest.

An interesting approach to PMP comprehension in marine mammals could be to determine whether, as in humans, diving perturbances can affect the production of PMPs. A similar study has previously been carried out measuring MP in the blood of Steller sea lions, needing more research to elucidate whether decompression has effects on its production (13).

In human divers, a fast ascent from the depth to the surface causes decompression sickness. At high pressures nitrogen is more soluble and accumulates dissolved in blood and tissues. During ascent nitrogen dissolved in the blood and tissues becomes less soluble forming bubbles as ambient pressure decreases swiftly (1). Studies have shown that even asymptomatic divers exhibit elevated levels of cellular microparticles in the blood, primarily carrying specific membrane proteins: CD41, CD31, CD66b, CD142, and CD235 (1). Blood levels of cellular MP have been demonstrated to be 2.4 to 11.7 times higher in symptomatic divers with decompression sickness compared to asymptomatic individuals (1). Therefore, levels of MP serve as reliable indicators for diagnosing and monitoring the progression of decompression sickness in humans (1). In fact, the decrease in MP levels correlates proportionally with the remission of the disease when treating decompression sickness (14). While decompression sickness in marine mammals is unclear, compatible lesions have been detected in some individuals (15, 16). The myoglobin content exhibited a positive and significant correlation with maximum dive duration in odontocetes, indicating its role in facilitating prolonged dives. Additionally, the syndrome of decompression sickness is intricately linked to the diving behavior of cetaceans, with variations in diving types influencing the likelihood of experiencing this condition (17). The rise in human life expectancy has led to an unprecedented increase in the population, consequently driving up the demand for food products such as fish. As a result, fishing practices have become industrialized and expanded into new areas, covering more than 55% of the ocean and causing overexploitation of certain fish populations (18, 19). This expansion has heightened interactions between fishermen and marine mammals, leading to competition for the same resources (20). Occasionally, marine mammals directly encounter fishing nets and become entangled, a process known as bycatch, where they are subsequently released back into the sea (21, 22). Bycatch may inflict severe injuries or mortality on the animals, potentially impacting their demographics and overall survival (23, 24). While marine mammals are anatomically and physiologically adapted to withstand normal diving conditions without experiencing decompression sickness, it has been hypothesized their adaptive mechanisms could fail in highly stressful situations like interaction with fisheries interaction or exposure to high intensity noise exposure (25, 26). There is an hypothesis that provides avenues for new areas of research, offers an explanation for how sonar exposure may alter physiology causing gas embolism, and provides a new mechanism for how air-breathing marine vertebrates usually avoid the diving-related problems observed in human divers (26). Severe gas embolism has been proposed to cause the death of the animals and eventual stranding on the coast (15, 16, 27). In such cases, the absence of bacteria or autolytic changes serves as histological indicators of pre-mortem gas bubble formation (27).

Additionally, a specific acoustic phenomenon termed “rectified diffusion” has the potential to directly cause gas embolism (15). Indeed, instances of mass strandings of marine mammals in regions characterized by high levels of noise pollution have been documented (28, 29). Notably, the significant stranding events involving beaked whales occurring mere hours or days following the use of military sonars in the same maritime zones stand out as crucial examples of the consequences of high intensity underwater noise on certain marine mammals, with gas embolism identified as the primary cause of death in some species (15, 30). Beaked whales typically engage in deep dives (31), so an acute stress response triggered by sonar signals has been proposed can result in gas embolism.

To study the role of the pathologies or anthropogenic threats in PMP production alterations the first step is to set up an assay for PMP detection. In the realm of marine mammals, the requisite methodology for analyzing PS exposure, MP formation, and detecting MP levels had not been fully developed. There is only one study on Steller sea lions that examines the measurement of blood microparticles to understand their correlation with decompression stress (13). However, our study specifically targets PMP since they constitute the predominant type in the bloodstream. Our group employed a flow cytometric technique utilizing an anti-human CD41 antibody, previously established and described in our prior research involving bottlenose dolphins, beluga whales, walruses, sea lions, and seals (32), the binding of the antibody to platelets is demonstrated not only by the morphological characteristics of the CD41+ events but also by their physiological response to the platelet agonist, showing the changes in annexin V exposure and production of PMP (32).

PMP hold promise as potential indicators of several diseases such as rheumatoid arthritis, thrombocytopenia or arterial thrombosis among others. It may also help to elucidate the pathophysiology of the alterations that occur during decompression processes in marine mammals, especially in captured or stranded animals. The primary challenge with these wild animals is accessing their blood when they are stranded, as they are often dehydrated or appear dead. This poses a significant problem as it is difficult to obtain a sufficient quantity of blood, or none at all in the case of dead animals. Additionally, another challenge is the necessity to process the blood samples within a narrow timeframe of 3–4 h, complicating the organization of the experiment. On the other hand, it provides a novel avenue for investigating platelet function in these species. Assessing real-time PS exposure and PMP production could potentially benefit veterinary practices in aquariums and research of marine mammals. In this work we present a pilot study on the effects of aspirin on the PMP production of dolphins, demonstrating the usefulness of the assay in the research on physiology and toxicological approaches in these species.

## 2 Materials and methods

### 2.1 Animals and samples

To carry out the study, 11 samples were obtained from 11 bottlenose dolphins (*Tursiops truncatus*; 1 sample per animal), 11 samples from 3 beluga whales (*Delphinapterus leucas*), 12 samples from 3 pacific walruses (*Odobenus rosmarus divergens*) and 4 samples from 4 Patagonian sea lions (*Otaria flavescens*; 1 per animal). All the animals inhabit Oceanogràfic Aquarium of the City of Arts and Sciences (Valencia, Spain) except two sea lions from Mundomar Aquarium (Benidorm, Spain). The samples were analyzed to evaluate PMP blood levels and real time PS exposure and release of PMP after activating the platelets with an agonist. All the experiments were approved by the Animal Care and Welfare Committee of the Oceanogràfic and Mundomar Aquariums (Reference: OCE-6-17).

### 2.2 Blood sampling

We collected 1 mL citrated whole blood from healthy animals that had been all previously trained to cooperate voluntarily with trainers and veterinarians for blood collection. In cetaceans, blood samples were drawn from a vein on the ventral surface of the caudal fin, while in pinnipeds, blood was obtained from interdigital veins on the caudal flippers. Specific equipment was utilized for blood sampling in both pinnipeds and cetaceans. This included a 21G gauge size Butterfly needle known as Venofix^®^, manufactured by Fa. Braun, which is commonly employed. Furthermore, single-use syringes with 10 mL capacity from Covetrus were used for blood collection in both pinnipeds and cetaceans. These syringes are designed for one-time use, ensuring sterility and minimizing the risk of contamination during the blood sampling process.

Samples were analyzed in the Cytomics Laboratory at the Príncipe Felipe Research Center (CIPF, Valencia, Spain) within 2 h after being obtained.

### 2.3 Reagents and solutions

Antibody CD41 PE, clone P2 was from Beckman Coulter (Cat. No: A07781). Annexin V was from the Annexin V-FITC / PI Kit, Miltenyi Biotec, (Order no. 130–092-052). Calcium ionophore A23187 (Merck, Cat No: C7522-1MG) was prepared at 10 mg/mL in DMSO, aliquoted and stored at −20°C. Modified Tyrode’s Buffer was home prepared using 137 mM NaCl, 2.8 mM KCl, 1 mM MgCl_2_, 12 mM NaHCO_3_, 0.4 mM Na_2_HPO_4_ and 10 mM HEPES, adjusted to pH 7.4 and stored at 4°C. Before starting each experiment, 0.35% Bovine Serum Albumin (BSA) and 5.5 mM glucose were added to the buffer and kept at room temperature until use. Annexin binding buffer was a modified Tyrode’s Buffer enriched with 0.22 mg/mL CaCl_2_.

### 2.4 Sample staining

Citrated whole blood was diluted 1:10 in Modified Tyrode’s Buffer. Then, 100 μL of the dilution was incubated for 10 min with 20 μL of the CD41-PE anti-human antibody, 2 μL of Annexin V-FITC and 150 μL of Annexin binding buffer at 37°C and 5% CO_2_. After that, 2 mL of Annexin binding buffer was added to each tube. Finally, 500 μL of the suspension was dispensed into an Eppendorf microtube for its acquisition on the flow cytometer.

### 2.5 Flow cytometry setup

The experiments were performed on a CytoFLEX S Flow Cytometer (Beckman Coulter, United States) using the cytometer-interfaced CytExpert software (Beckman Coulter, Californa, United States).

The flow cytometer was set up to measure Forward Scatter signal (FSC), Violet Side Scatter signal (VSSC), Annexin-V-FITC fluorescence (FL1, exc 488 nm/em 525 nm), CD41-PE fluorescence (FL10, exc 561 nm/em 585 nm) and time. To optimize the detection of PMP, we used the SSC signal from the violet laser (405 nm, VSSC), since this signal facilitates the amplification of the differences in the refractive indices between the particles and their surrounding medium. The trigger used for PMP assessment was VSSC-Height, with a threshold in 40,000. All the signals were acquired in logarithmic amplification. Data analysis was performed using CytExpert Software (Beckman Coulter) and FlowJo^™^ v10.5.3 Software (BD Life Sciences).

#### 2.5.1 Real time PS exposure and PMP production evaluation stimulating platelets with calcium ionophore A23187

**Figure 1:**
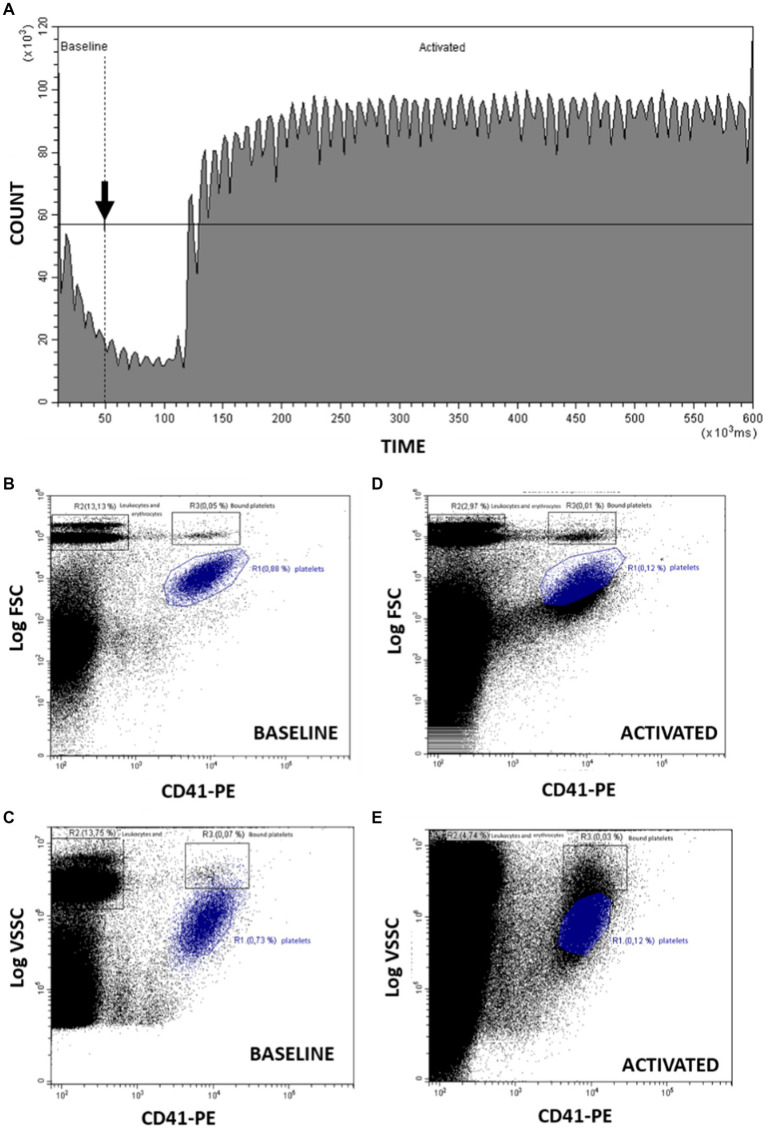
Analysis by flow cytometry of PMP formation stimulating platelets with Ca^2+^ ionophore A23187. **(A)** Count of cells and time course representative of the kinetics before (baseline) and after (activated) adding the stimulus (arrow). The observed periodic oscillations in the Count vs. Time graph reflect the minor variations in sampling rate provided by the peristaltic pump of this flow cytometer model. **(B)** From “baseline,” dot-plot CD41-PE/FSC for identification of platelets (R1), erythrocytes and leukocytes (R2) and coincident platelets erythrocytes and leukocytes (R3). **(C)** From “activated,” dot-plot CD41-PE/FSC changes after platelet activation with A23187. **(D)** From “baseline,” dot-plot CD41-PE/VSSC for identification of platelets (R1), erythrocytes and leukocytes (R2) and coincident platelets erythrocytes and leukocytes (R3). **(E)** From “activated,” dot-plot CD41-PE/VSSC changes after platelet activation with A23187. R1: region including platelets; R2: region including erythrocytes and leukocytes; R3: region including platelets bound to or coincident in flow with erythrocytes and leukocytes.

A protocol previously described in humans (32) was adapted and modified to marine mammals. Figure 1A shows the count of cells and time course representative of the kinetic evaluated in the study. “Baseline” region corresponds to the time before adding the stimulus until 50 s of acquisition. At this time the calcium ionophore A23187 is added to the sample while the cytometer continues aspirating the sample. “Activated” region spans from the addition of the agonist to the end point and includes the platelets that have been stimulated.

Platelets and PMP can be identified using the CD41-PE fluorescence combined either with FSC or VSSC signals, using size or internal complexity, respectively, as morphological indicators (Figures 1B–E). Figure 1B shows the resting platelet population identified as positive CD41 events with the smallest relative size (region R1). Erythrocytes and leukocytes are also distinguished as the larger CD41 negative events (region R2). On the other hand, R3 include platelets bound to with erythrocytes or leukocytes or coincident with them when crossing the cytometer laser. When platelets are activated with calcium ionophore 0.02 μg/mL A23187 starts the PS exposure and PMP production decreasing the FSC and CD41 signal as seen in Figure 1C. These changes can also be observed using VSSC instead of FSC as detailed in Figures 1D,E.

**Figure 2:**
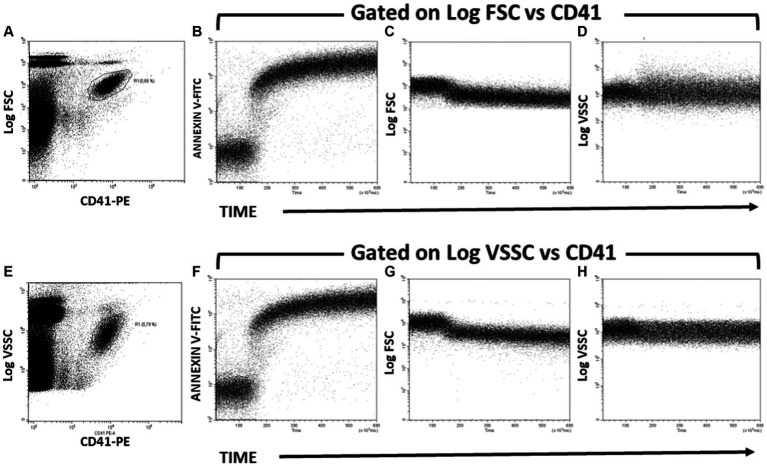
Kinetics of PS exposure and PMP formation after activating Bottlenose dolphin platelets in a whole blood sample with calcium ionophore A23187. **(A,E)** Gating of the main platelet population on CD41-PE/Log FSC **(A)** and CD41-PE/Log VSSC **(E)** dot plots for further kinetic representation of changes over time in Annexin-V-FITC fluorescence **(B,F)**, Log FSC **(C,G)**, and Log VSSC **(D,H)** after addition of calcium ionophore A23187 50 s after the start of the run. The kinetic plots in panels **B–D** are gated in the platelet region defined in the dot plot of panel **A**. The kinetic plots in panels **B–D** are gated in the platelet region defined in the dot plot of panel **B**.

Kinetics of PS exposure and PMP formation after activating the platelets with A23187 were assessed following the Annexin V-FITC and FSC signals over time (Figure 2). These changes can also be assessed by identifying platelets from CD41/FSC (Figures 2A–D) or CD41/VSSC (Figures 2E–H) dot plots. Briefly, the baseline Annexin V-FITC and FSC signals were registered for 20–50 s. At this time, calcium ionophore was added to the sample in acquisition and mixed with a Pasteur pipette, while the cytometer continued aspiring the sample. Changes over time on Annexin V-FITC fluorescence intensity and FSC and VSSC signals were recorded up to 10 min.

#### 2.5.2 Obtaining numerical values of platelet PS exposure and PMP formation

**Figure 3:**
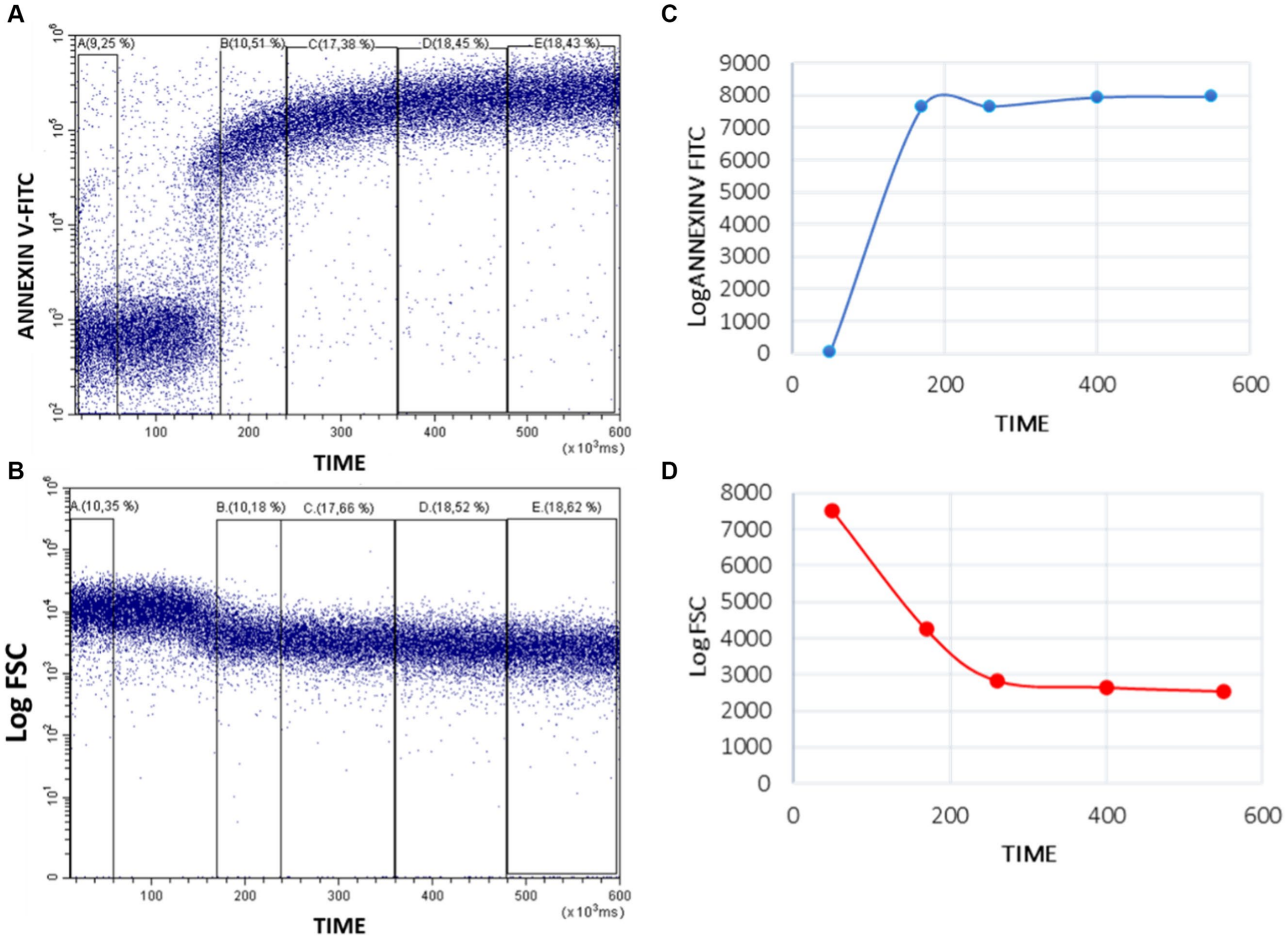
Quantitative analysis of Annexin-V-FITC binding and FSC decrease in whole blood platelets after activating Bottlenose dolphin platelets in a whole blood sample with calcium ionophore A23187. Five regions **(A–E)** were defined over the Time axis in Annexin V-FITC/Time **(A)** and FSC/Time kinetic plots **(B)** and the mean intensity values obtained with the CytExpert software. The resulting values were represented in standard kinetic graphs displaying in arbitrary units (Y-axes) the mean fluorescence intensity (Annexin-V-FITC) **(C)** and the mean scatter intensity (Log FSC) variations along time **(D)**.

The analysis was carried out with the Flowjo software using the same strategy for region selection as in the sample acquisition. Changes in Annexin V and FSC signals over time were represented in dot plots. To obtain numerical parameters, analytical regions were defined throughout the length of the plot, dividing them in five parts to evaluate the mean fluorescence intensity (MFI) of Annexin V and FSC (A to E regions) variations over time (Figure 3). In the Annexin V/Time dot plot, region A corresponds to the baseline fluorescence intensity of Annexin V-FITC in resting platelets. After stimulating with calcium ionophore, the Annexin V-FITC MIF increases, due to the externalization of PS in activated platelets. Annexin V binds to PS increasing its fluorescence. The peak of fluorescence is framed in region B. Regions C to E record the changes in PS exposure over time until 10 min of acquisition (Figure 3A). In FSC/time dot plot, region A establish the baseline platelets relative size. After stimulating with calcium ionophore, the platelets break down into PMP, so FSC signal progressively decreases over time (regions B to E; Figure 3B).

From these regions we propose different parameters to evaluate PS exposure and PMP production:

Focusing on Annexin-V/time dot plot, we obtained the mean of the time and Annexin V-FITC MIF (fluorescence arbitrary units, FAU) for each region (A–E). These values can be represented in a graphic (Figure 3C). From these, we calculate: Ratio between peak and baseline Annexin-V fluorescence intensity (RPB): the fold that platelet PS exposure increases after A23187 addition; differential between peak and baseline Annexin-V fluorescence intensity (ΔPB): the absolute difference in platelet PS exposure between activated and resting platelets; ratio between end-point and baseline Annexin-V fluorescence intensity (REB): the fold that platelet PS exposure remains elevated above the PS exposure in resting platelets; differential between end-point and baseline Annexin-V fluorescence intensity (ΔEB): the absolute difference between the remaining platelet PS exposure at 10 min post-activation and in resting platelets; and slope of the Annexin-V fluorescence curve from the peak to the end point (SPE): the rate of PS exposure after platelet stimulation.

In the FSC/time kinetics plot, for each region (A to E), we obtained the mean FSC and time. These values can be graphed (Figure 3D) and the slope of the line could be calculated to quantify the decrease speed of the relative size after PMP formation (SFSC).

### 2.6 Obtaining normal values of PS exposure and PMP formation for the different species

To obtain the values of PS exposure and PMP production in healthy individuals of the different species, we analyzed RPB, ΔPB, REB, ΔEB, SPE and SFSC in 11 dolphins (1 sample per animal), 11 samples from 3 beluga whales, 12 samples from 3 walruses and 4 samples from 4 sea lions. Calculating the mean, median, standard error (SEM), minimum and maximum for each parameter in all the species.

### 2.7 Application of PS exposure and PMP formation assessment in *in vitro* toxicological studies in marine mammals

Blood from three healthy dolphins was used for this proof of concept of the effects of aspirin on PMP production in dolphins.

#### 2.7.1 Preparation of aspirin stock and culture medium

The final aspirin concentrations used in the assay were 0.02 μM, 2 μM, and 200 μM. Aspirin stocks were prepared each day solubilizing it as ethanol solutions at concentrations 200 times higher than those used in the assay. For each experiment, the aspirin stocks were diluted in modified culture medium obtaining the double the required final concentration (2X) per well.

Culture medium was RPMI 1640 + GlutaMAX-I (GIBCO, 61870–010) supplemented with 10 mM HEPES (GIBCO, 15630–056), 0.1 mM non-essential amino acids (GIBCO, 11140), 50 U/mL penicillin/50 μg/mL streptomycin (GIBCO, 15140–122), 50 μM 2-mercaptoethanol (GIBCO, 21985–023) and 10% bovine serum (GIBCO, 26010–074).

#### 2.7.2 Blood treatment with aspirin

For the *in vitro* studies, 96-well plates were used for acute and sustained (24 h) blood treatment with different concentrations of aspirin. To do this, 50 μL of citrated whole blood, 50 μL of modified RPMI medium and 100 μL of 2X aspirin were mixed in each well and incubated up to 24 h. PMP production was evaluated at different concentrations of aspirin comparing with the non-exposed control.

On the other hand, the acute response to aspirin was also studied by exposing the blood to the compound and measuring its immediate effects, without previous incubation.

### 2.8 Statistical analysis

The statistical significance of the differences in PS exposure and PMP formation parameters between animals per sex, age or species, were evaluated by a t-test in Graphpad Prism 5. In the toxicological study with aspirin, the effect of the drug in the PS exposure and PMP formation was statistically assessed using t-test and ANOVA in Graphpad Prism 5.

## 3 Results

The use for the first time of Annexin-V as a PS marker in platelets of marine mammals was successful. The combined measurement of Annexin-V-FITC, CD41-PE, FSC and VSSC signals was useful to discriminate PMP in marine mammals, as previously described for humans (33). Platelets response to stimulation with the agonist was similar to that observed in humans too, increasing the MFI of Annexin-V by PS exposure and reducing FSC signal by PMP formation (33) (Figure 2).

### 3.1 Measurement of PS exposure and PMP production in stimulated platelets

The human-reacting monoclonal antibody CD41 (clone P2) was found to identify the platelets in different species of marine mammals (32), but also PMP. PMP are platelet membrane fragments that can be distinguished by their expression of CD41, PS and their low relative size. Our group has been exploring a technique utilizing flow cytometry that shows promise in distinguishing PMP in marine mammals (32).

Figure 1A shows a time course representative of the kinetic experiments performed in this work. As described in point 2.5.1 of Material and Methods, fluorescence and light scatter signals are recorded continuously up to 600 s. In our experimental setup, the Ca^2+^-ionophore A23187, a strong platelet agonist, is added to the sample vial 50 s after the start (arrow Figure 1A), while the cytometer continues aspirating the sample. The cytometer software allows to define two consecutive regions in the count-versus-time graph that include the events registered before (Baseline) or after (Activated) the addition of the stimulus. Such regions can be used to gate in separate plots the basal cytometric features of the unstimulated sample (panels Figures 1B,D) and the changes induced by the Ca^2+^-ionophore (panels 1C and 1E). With this approach, real-time monitoring of the platelet activation process is feasible.

Platelets in whole blood samples can be identified clearly by their expression of CD41, a constitutive marker of platelet membrane, and their morphological features estimated by their light scatter signature. Prior to ionophore addition, bivariate plots of CD41-PE vs. blue-laser forward scatter (Log FS, panel Figure 1B) or of CD41-PE vs. violet-laser side scatter (Log VSC, panel 1D) clearly show the cluster of individual platelets, as well as the cluster that represents the platelets that are bound-to or coincident-with other blood elements (mostly erythrocytes). More relevant to our experimental objective, the presence of circulating platelet-derived microparticles (PMP) is supported by a cohort of events characterized by decreased intensity of CD41-PE fluorescence and of either Log FSC or Log VSC, and that gradually merge into the background noise.

The bivariate plots gated in the time region post-ionophore addition allow to visualize the A23187-stimulated release of microparticles by activated platelets. Panel Figure 1D shows an average decrease of FSC intensity in the gated platelet population upon ionophore addition, accompanied of an increase in the continuum of lower CD41-PE/lower FSC events. The changes in VSSC and CD41-PE intensity (panel Figure 1E) upon ionophore addition are less straightforward. As seen in the plot, the gated platelet population maintains the average VSSC, while a continuum of CD41-PE+/higher VSSC events reaches and overpopulates the plot area where platelets bound or coincident with blood elements are expected. This observation suggests that this new population reflects the swarm effect (34) due to simultaneous laser illumination of platelets and released PMPs, amplified by the higher sensitivity of VSSC for assessing particle complexity by flow analysis (35). On the other hand, consistent with the changes depicted in panel Figure 1D, an increase in the continuum of lower CD41-PE/lower VSSC events is also observed, confirming that VSSC is also suitable for detecting PMP shedding.

Finally, it is worth to mention that the shedding of PMP induced by the ionophore explains the fast and noticeable increase in event count rate happening in the ungated kinetic plot (panel Figure 1A). As clearly seen, there is a sharp increase (approx. seven-fold) in the count rate that reflects the rapid appearance of PMP in the sample followed by stabilization of the count rate until the end of the run. This is consistent with the immediate effect of the strong platelet agonist used and shows that this assay may provide a very fast endpoint for assessing platelet functional responses associated to experimental or clinical studies of hemostasis.

Activated platelets expose PS, thus providing the procoagulant surface to which thrombin-generating enzyme complexes bind and assemble. The exposure of PS on platelets can be assessed in flow by the binding of fluorochrome-labeled Annexin V to platelets (36), while the subsequent release of PMP upon platelet activation can be monitored by the emerging of Annexin V-positive events with lower FSC or VSSC signals than the main platelet population. Since the generation of PMP by whole blood platelets induced by exposure to Ca^2+^-ionophore is a very fast process (Figure 1) we attempted to monitor in real time the generation of platelet procoagulant surface. To this extent, whole blood samples were stained with both CD41-PE (for platelet and PMP identification) and Annexin V-FITC (for detecting PS exposure in platelets and PMP), and then challenged with Ca^2+^-ionophore A23187 in the same experimental conditions as indicated for Figure 1. Again, the main platelet population was identified and gated in CD41-PE vs. Log FSC (panel Figure 2A) or CD41-PE vs. Log VSSC (panel Figure 2E) bivariate plots and the variations in Annexin V-FITC (panels Figures 2B,F), Log FSC (panels Figures 2C,G) and Log VSSC (panels Figures 2D,H) monitored by means of kinetic graphs gated-in by the indicated criteria.

As seen in Figure 2, addition of Ca^2+^-ionophore A23187 induced a very fast binding of Annexin to platelets, as evidenced by the time course of the kinetic plots of Annexin V-FITC versus Time (panel Figures 2B,F), which were quite similar to the time course of the Count versus Time graph in Figure 1, thus supporting that Ca^2+^-ionophore stimulation of platelets results in an almost simultaneous generation of procoagulant surface and PMP shedding, as expected (36). In consistence, the increase in Annexin V binding overlaps essentially in the time interval with a clear decrease of FSC signal (panels Figures 2C,G) and a less pronounced decrease of VSSC (panels Figures 2D,H). No apparent difference was observed in these kinetics regarding whether the platelet population was gated by its Log FSC (panel Figure 2A) or Log VSSC (panel Figure 2E) signatures.

**Figure 4:**
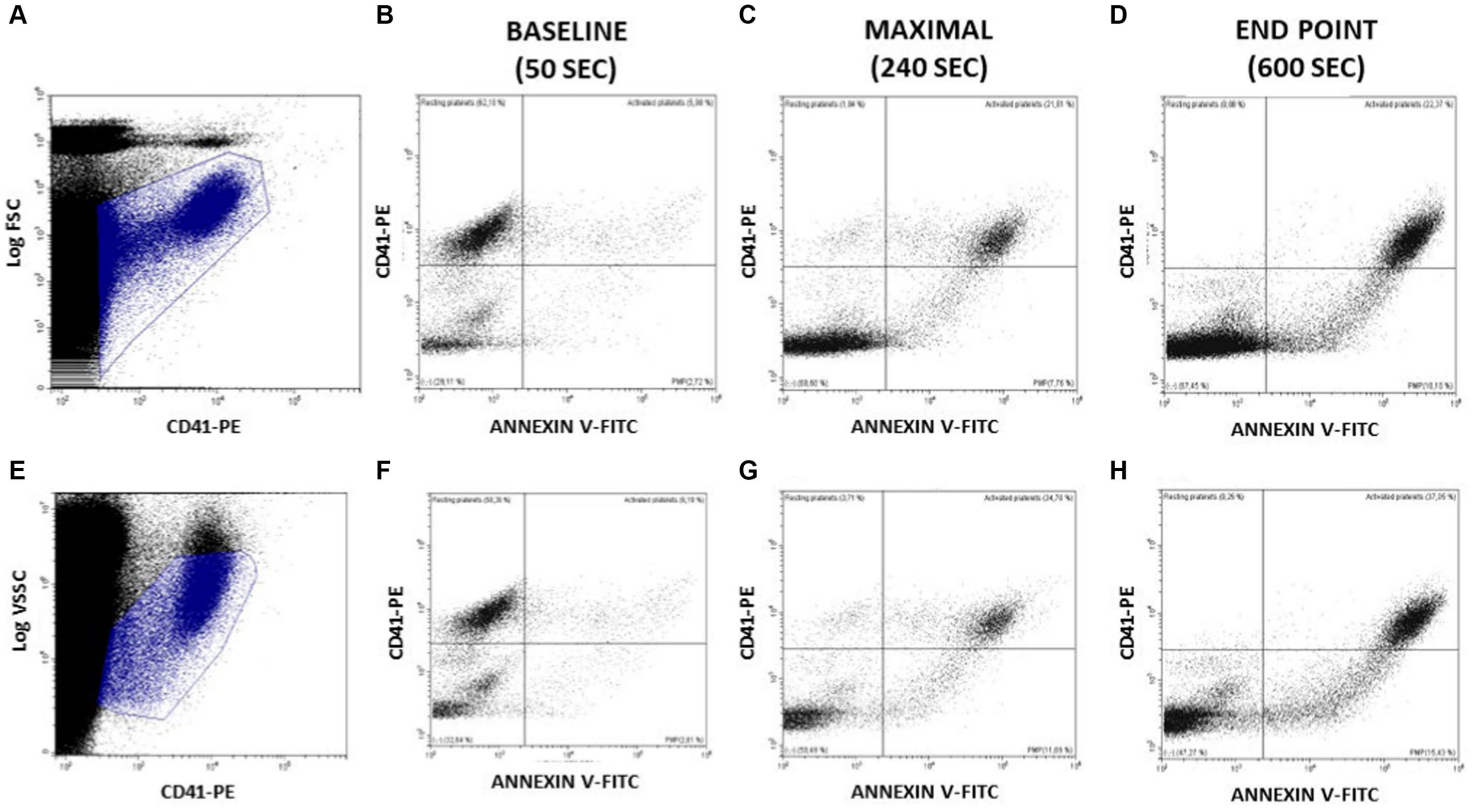
Assessment of PS exposure on platelet surface and PMP generation at relevant times after activating Bottlenose dolphin platelets in a whole blood sample with calcium ionophore A23187. The main platelet population and the continuous cohort of PMPs were gated for further analysis in the displayed regions amply drawn on CD41-PE/Log FSC **(A)** or CD41-PE/Log VSSC dot plots **(E)**. At the indicated time intervals a series of CD41-PE/Annexin V-FITC dot plots were used to display the relative proportions of platelets at rest (CD41+/Annexin V- events); platelets exposing PS (CD41+/Annexin V+ events); PMP of varying size and PS (CD41 variable/Annexin V variable events) and PMP merging with background noise (CD41−/Annexin V- events) **(B–D** and **F–H)**.

In order to complete the above observations with a more accurate description of PMP generation (Figure 4), we defined two additional gating criteria, based on CD41-PE vs. Log FSC (panel Figure 4A) or CD41-PE vs. Log VSSC (panel Figure 4E) enlarged regions that could allow to track events described by their CD41-PE and Annexin V-FITC expression in a more extended range. This strategy displays better the populations of non-activated (CD41+/Annexin V-) and activated (CD41+/Annexin V+) platelets, and the cohort of generated PMP with different sizes, as estimated by their variable expression of both CD41-PE and Annexin-V (CD41var/Annexin Vvar). The CD41neg/Annexin Vneg region includes the background noise and the undetectable PMP beyond the limit of sensitivity of the flow cytometer. Although we have not made still any attempt to calibrate the size of PMP nor to quantify their abundance, we show in Figures 4B–D,F–H, the distribution of the above populations in three distinct relevant stages of the ionophore-activation experiment, namely prior to ionophore addition (baseline: 0–50 s); at the time of maximal Annexin V-FITC binding (maximal: 120–240 s) and at the final time of the run (end point: 460–600 s). As seen, at baseline most platelets are non-activated, but the presence of some activated platelets and PMP is observed. At maximal, most platelets are activated and PMP are increased, but some platelets are unstimulated or still express lower levels of Annexin V-FITC. At the end point, no resting or partially activated platelets are evident and the cohort of PMP is more abundant. As in the previous experiments, quite similar distributions were observed, independently of whether Log FSC or Log VSSC were used as gating criteria.

**Figure 5:**
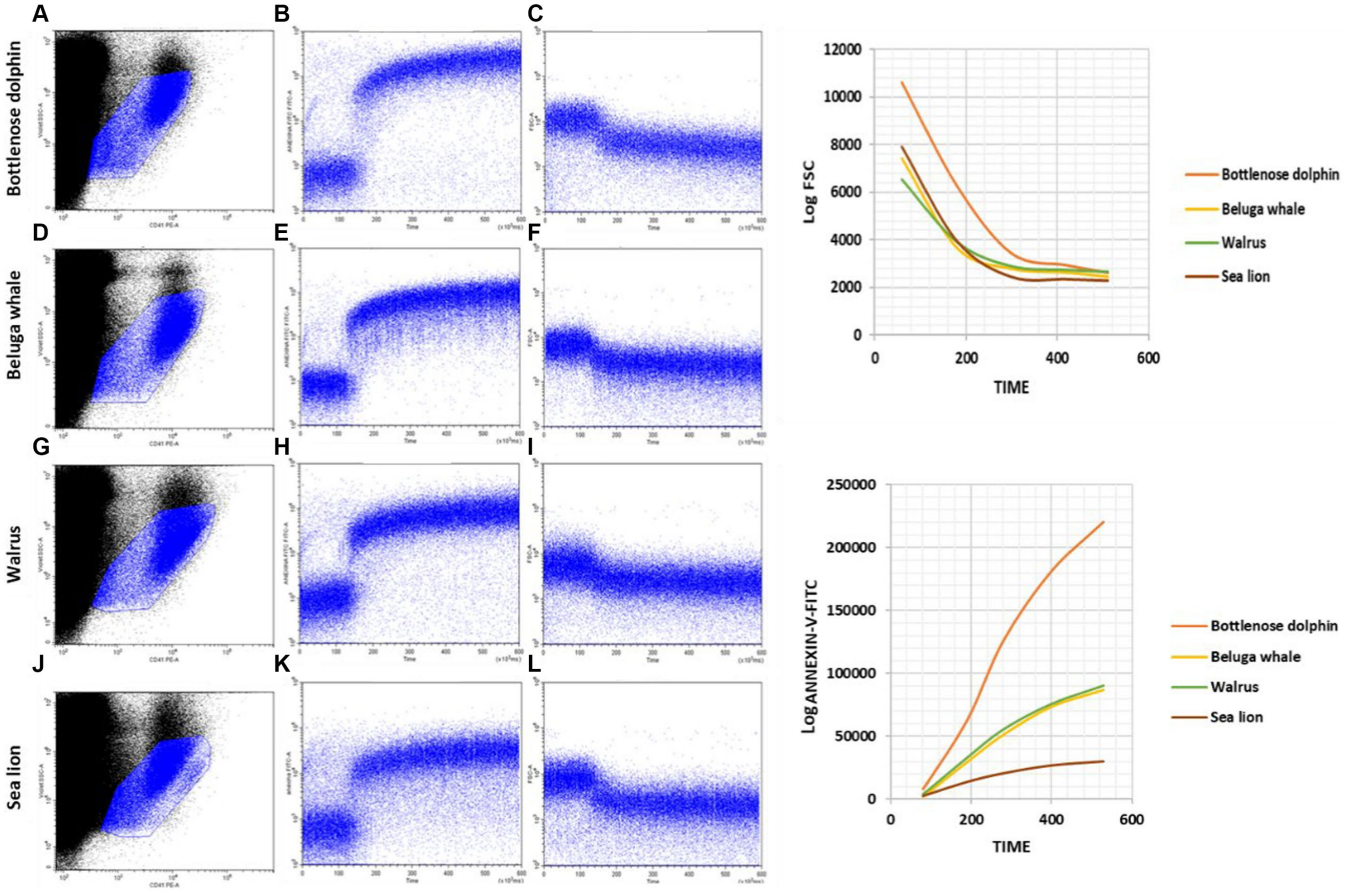
Kinetics of PS exposure and FSC decrease after activating marine mammal platelets in whole blood samples with calcium ionophore A23187. **(A,D,G,J)** Gating of the main platelet population on CD41-PE/Log VSSC dot plots for further kinetic representation of changes over time in Annexin-V-FITC fluorescence **(B,E,H,K)** and Log FSC **(C,F,I,L)** after addition of calcium ionophore A23187 50 s after the start of the run. Plots are representative examples of the results obtained with Bottlenose dolphins **(A–C)**; Beluga whales **(D–F)**; Walruses **(G–I)** and Sea lions **(J–L)**.

Our kinetic approach based on Annexin V binding and FSC variations can be made quantitative by establishing appropriate intervals in the Annexin V-FITC versus Time (Figure 3A) and the Log FSC versus Time (Figure 3C) plots and calculating the mean intensity of fluorescence and FSC signals, that can be then plotted in conventional graphs (Figures 3B,D). Such graphs allow to compare better different processes of platelet activation or to show differences among different individuals or among zoological groups. In this context, we have extended our kinetic assay, that has been set up using blood samples from bottlenose dolphins to individuals of other species of marine mammals, namely beluga whales, walruses and sea lions. As seen in Figure 5, the kinetic assay here described can be applied without any modification of the experimental conditions and cytometer settings to other marine mammals and shows quite similar qualitative behavior of platelets when challenged with the Ca^2+^-ionophore A23187 used for activation of bottlenose dolphin platelets.

### 3.2 Normal values of PS exposure and PMP formation in the different species and differences by age, sex or specie

The median, standard error (SEM), minimum and maximum values for each parameter studied of PS exposure and PMP formation in bottlenose dolphins, beluga whales, walruses and sea lions are detailed in Tables 1–4.

**Table 1:** Range of values for each PS exposure and PMP formation parameters in dolphins under human care.

	SFSC	RPB	REB	ΔPB	ΔED	SPE
Bottlenose dolphins *n* = 11, 11 samples	−18.5 ± 1.5 (min: −27; max: −12)	41.7 ± 15.6 (min: 1.5; max: 182)	19.5 ± 5 (min: 1.6; max: 51)	23.932 ± 2.876 (min: 11271; max: 41804)	16.809 ± 1.326 (min: 8601; max: 23384)	13 ± 5.6 (min: −38; max: 33)
Calves *n* = 3	−15 ± 1.8	77 ± 35	35 ± 15	25.890 ± 4.885	19.316 ± 1.170	25 ± 8
Adults *n* = 11	−19 ± 1.3	23.7 ± 5.6	23.5 ± 5	20.835 ± 2.124	19.322 ± 1.666	22.5 ± 3
Calves vs. Adults	NSD	p = 0.02*	NSD	NSD	NSD	NSD
Males *n* = 5	−17.8 ± 1.5	57 ± 23	28 ± 9	24.378 ± 2.808	19.116 ± 814	23 ± 4.6
Females *n* = 9	−16.7 ± 1.4	23 ± 7	25 ± 6	20.552 ± 2.622	19.434 ± 2042	23 ± 4
Males vs. Females	NSD	NSD	NSD	NSD	NSD	NSD

Significant differences were observed between calves and adults in the PS exposure after stimulation with calcium ionophore A23187 (*p* ≤ 0.05*) (NSD: *p* ≥ 0.05).

**Table 2:** Range of values for each PS exposure and PMP formation parameters in beluga whales under human care.

	SFSC	RPB	REB	ΔPB	ΔEB	SPE
Beluga whales *n* = 3, 11 samples	−12 ± 0.5 (min: −14; max: −9)	63 ± 14 (min: 3.3; max: 163)	29.5 ± 4 (min: 15; max: 60)	24.055 ± 3.335 (min: 1142; max: 42076)	12.248 ± 1.190 (min: 5289; max: 17256)	−2 ± 5 (min: −25; max: 26)
Adults *n* = 2, 8 samples	−13.00 ± 0.3	74.16 ± 17.7	32.93 ± 5	27.367 ± 3.241	13.343 ± 1.317	−4.3 ± 5.6
Calf *n* = 1, 3 samples	−9.667 ± 0.3	33.44 ± 15	20.64 ± 3.7	15.224 ± 7.083	9.329 ± 1915	4.333 ± 11
Calf vs. Adults	*p* = 0.0003***	NSD	NSD	NSD	NSD	NSD
Males *n* = 2, 6 samples	−11.50 ± 0.8	47.41 ± 13.5	28.72 ± 4.7	20.608 ± 5.456	12.380 ± 1.696	4.5 ± 6.2
Female *n* = 1, 5 samples	−12.80 ± 0.5	81.83 ± 26.3	30.61 ± 7.6	28.191 ± 2.840	12.090 ± 1841	−9.8 ± 7
Males vs. female	NSD	NSD	NSD	NSD	NSD	NSD

Significant differences were observed between the calf and adults in the rate of decrease in the FSC signal after stimulation with A23187 (*p* = 0.0003***) (NSD: *p* ≥ 0.05).

**Table 3:** Range of values for each PS exposure and PMP formation parameters in walruses under human care.

	SFSC	RPB	REB	ΔPB	ΔEB	SPE
Walruses *n* = 3, 12 samples	−11.6 ± 0.7 (min: −15; max: −7)	71.7 ± 17.5 (min: 13; max: 226)	39.7 ± 4 (min: 13; max: 67)	26.019 ± 3.171 (min: 11247; max: 41778)	18,320.5 ± 2.622 (min: 9021; max: 37630)	14.6 ± 7.8 (min: −25; max: 51)

All the animals were adult females.

**Table 4:** Range of values for each PS exposure and PMP formation parameters in sea lions under human care.

	SFSC	RPB	REB	ΔPB	ΔEB	SPE
Sea lions *n* = 4, 4 samples	−18.7 ± 3 (min: −27; max: −14)	22 ± 8 (min: 5; max: 43)	30 ± 9 (min: 7; max: 46)	14.005 ± 1976(min:9664; max: 17883)	20.344 ± 3.980 (min: 15431; max: 32149)	31.75 ± 10 (min: 18; max: 62)

All the animals were adult males.

Within the same species, differences in platelet PS exposure and PMP production were only found between cetaceans of different ages. To confirm these results, the population size should be increased.

Regarding differences by species, our findings are shown in Table 5. The fast and sustained increased in Annexin V-FITC binding is observed in dolphins’ samples (Figure 5). However, when such individual cytometric plots are transformed into quantitative standard kinetic graphs, a better comparison among the rate and intensity of the parameters reporting platelet activation is possible. Thus, the strongest expression of procoagulant surface is observed in bottlenose dolphin, while sea lion shows the lowest velocity and intensity of expression. Beluga whale and walrus have a similar behavior among them, with velocity and intensity values intermediate between bottlenose dolphin and sea lion. As for the decrease in FSC the intensity of the change is highest in bottlenose dolphin (Table 5), but sea lion and beluga whale, which have similarly smaller platelet FSC signals than dolphins, show similar rates of FSC decrease among them and compared to dolphins. Finally, walrus shows both slightly lower FSC baseline values and rate of FSC decrease. However, walruses exhibited a significantly higher end-point PS concentration than dolphins (REB).

**Table 5:** Differences in PS exposure and PMP formation between species with ANOVA (NSD: *p* ≥ 0.05).

	SFSC	RPB	REB	ΔPB	ΔEB	SPE
Dolphins vs. beluga whales	D > BW *p* ≤ 0.01**	NSD	NSD	NSD	NSD	NSD
Dolphins vs. Walruses	D > W *p* ≤ 0.01**	NSD	W > D *p* ≤ 0.01**	NSD	NSD	NSD
Beluga whales vs. Walruses	NSD	NSD	NSD	NSD	NSD	NSD

** SFSC was significant different in dolphins than in beluga whales and walruses (**p ≤0.01) and REB was significantly higher in walruses than in dolphins (**p ≤ 0.01) (NSD: *p* ≥ 0.05).

### 3.3 Application of PS exposure and PMP formation assessment to *in vitro* toxicology assays

The assay adapted in this work to measure platelet PS exposure and PMP formation has been successfully applied to *in vitro* toxicology studies. Specifically, aspirin had effects on both processes in dolphin platelets.

#### 3.3.1 Acute exposure to aspirin

PMP formation rate after the stimulation with A23187 was significantly lower in the samples treated with aspirin than in controls. This is evident by measuring the speed at which the FSC signal decreases when passing from platelets to PMP. Specifically, the production of PMP was 50% ± 18.5 slower in samples exposed to 0.02 μM than in controls (*p* = 0.036*) and 38% ± 0.06 slower in samples exposed to 2 μM (*p* = 0.0006***) than in controls. At 200 μM, the speed was reduced by 40%, although not significantly, due to the high variability of the data.

PS exposure, given by changes in Annexin-V fluorescence, was not affected by acute aspirin exposure at any concentration, with no significant differences comparing to the control.

#### 3.3.2 24 H exposure to aspirin

**Figure 6:**
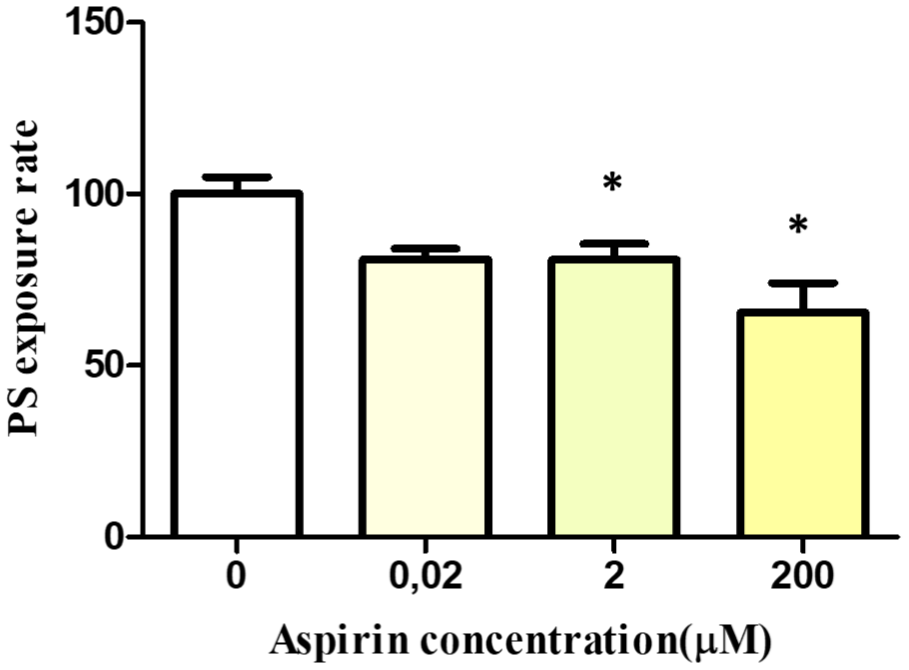
*In vitro* effect of aspirin on platelet PS exposure and PMP formation in whole blood platelets of Bottlenose dolphins after activation with calcium ionophore A23187. Dose–response of PS exposure rate in whole blood platelets after 24 h of incubation with aspirin.

After 24 h of incubation, PS exposure rate after stimulation with A23187 decreased in the samples treated with the highest aspirin concentrations. Specifically, it decreased by 19.2 ± 4.6% at 2 μM (*p* = 0.04) and 34.5 ± 8.5% at 200 μM (*p* = 0.02). No significant differences were observed between the control and the sample treated with 0.02 μM (Figure 6).

However, PMP formation (given by FSC signal changes), was not affected by 24 h aspirin exposure at any concentration, with no significant differences comparing to the control.

## 4 Discussion

Platelet-derived microparticles (PMP) constitute a predominant type of microparticles in healthy animals, released during platelet activation, stress, or apoptosis (1–3). Physiologically, these particles are typically present in the range of 100–4,000 PMPs μL^−1^of blood in humans depending intrinsic characteristics of the flow cytometer and the calibration strategy that has been used (4). Changes in PMP levels have been associated with various health conditions, including decompression sickness, thrombocytopenia, rheumatoid arthritis, cancer, arterial thrombosis, atherosclerosis, immune thrombocytopenic purpura, and malaria infection (1, 3, 6–10). On the one hand, elevated PMP levels are implicated in thrombus formation, while reduced levels are connected to bleeding tendencies observed in conditions like Castaman’s defect or Scott’s syndrome (3, 11).

Hence, monitoring blood PMP levels proves to be a valuable diagnostic and monitoring tool for various disorders. While some of these conditions are not extensively documented in marine mammals, this is likely due to limited available information rather than a lack of affliction. The challenges of accessing sea creatures may contribute to this data gap. Establishing a method for detecting PMP in the blood of dolphins, beluga whales, walruses, and sea lions can enhance the diagnostic tool for clinical assessments across multiple pathologies. In this work we have developed the methodology to measure in real time the exposure of PS in platelets and the formation of PMP by stimulating platelets with an agonist. Furthermore, we have carried out a pilot study to obtain normal values in these parameters in each species and enable the early detection of alterations in this process, related to different disorders, stress, pollution.

Although it is still an incipient field of study, PMP not only have been described as biomarkers of certain pathologies, but as triggers of others too (3), transporting growth factors, mRNA, microRNAs or surface receptors from platelets to other cells (37). PMP fuse with the target cell membrane and deposit the content (37), modulating multiple cell behaviors related to inflammation, immunomodulation, adaptative immunity, hemostasis, or tumor cell growth or metastasis (3). On this last case, PMP can be implicated in metastasis, but in the growth suppression of solid tumors too in mice and humans (38). They also play an important role in the course of diseases with endothelial injuries, such as some diabetic nephropaties (39) or atrial fibrillation (40), adhering to the subendothelium and stimulating the adhesion of other cells like platelets or leukocytes, leading to the formation of an atheroma and finally a local thrombus (3). In this context, PMP have been proposed as a possible therapeutic tool for the endothelium tissue repair (3). Despite this idea is already under investigation, the local application of these microparticles could be a new tool in the treatment of some vascular or brain injuries (40).

The study of PMP is expanding, revealing associations with various pathologies, and serving as enhancers, valuable biomarkers for diagnosis, and potentially even part of therapy. While much of the progress in understanding PMP is derived from research in humans and mice, this knowledge can be extrapolated to other mammalian species, including marine mammals. The initial step involves detecting PMP in their blood, utilizing established methodologies from human and mouse studies across all relevant species. In our work, we adapted this method for bottlenose dolphins, beluga whales, walruses, and sea lions. The selection of these species was intentional, given that marine mammals serve as sentinel species in marine ecosystem studies. Their considerable longevity and position at the apex of the food chain make them the ultimate recipients of numerous threats in the sea (13, 41). Using this assay in conservation studies we can also approach how different anthropogenic threats such as pollution, bycatch or maritime noise can affect the function of platelets and specifically the exposure of PS and formation of PMP.

Although the number of samples has not been too large, in this pilot study we show normal values of PS exposure and PMP formation in the different species under study, which can serve as a reference to detect alterations related to different pathologies or human impacts. From this information, we have also been able to observe that there are some differences in the behavior of platelets against A23187 between species, mainly the greater PS exposure of dolphin’s platelets comparing to the rest of the species.

The application of this methodology and establishing normal values for each species is an important novelty in the study of the physiology of these species. There is only a prior study in MP detection on Steller sea lions, specifically in connection with decompression stress (13). In this study, total blood MP levels were evaluated, using beads of known size as reference. However, no previous study in marine mammals has evaluated cell type-specific MP levels, such as PMP, or their real time formation. On the other hand, Fahlman et al. did not elucidate a way to use PMP levels as a biomarker of decompression alterations.

The relevance of decompression sickness in wild marine mammals remains under discussion. Intense stress responses at depth may compromise their adaptive diving mechanisms, potentially leading to gas embolism (26, 28). Developing a simple, non-invasive technique to assess PMP levels in marine mammals, similar to approaches used in humans (1), could significantly advance the diagnosis and understanding of decompression sickness in this taxonomic group. This advancement is crucial, especially since stranded animals often reach the coast in poor condition or deceased, and PMP detection could provide insights into their physiological status and the potential cause of stranding. Additionally, regularly assessing PMP levels in the blood during rehabilitation could offer a non-invasive means to gauge disease progression following a decompressive event. In humans, as the disease subsides, there is a concurrent decrease in PMP levels (1).

Definitely, this assay could be a diagnostic tool in processes with altered platelet function, using A23187, ADP or others as agonists. Our results show as have been explain in Results an average decrease of FSC intensity in the gated platelet population upon ionophore addition, accompanied of an increase in the continuum of lower CD41-PE/lower FSC events. These changes are compatible with a decrease in platelet size resulting from ionophore-triggered shedding of PMP from the platelet body (36). Platelets, upon activation, release components of their plasma membranes into the extracellular space, forming microparticles (MPs). It has been demonstrated that platelets release MP following activation with agonists such as collagen and/or thrombin, the Ca^2+^ ionophore A23187, or the complement protein C5b-9, which induces PMP formation. Therefore, our findings are consistent with previous findings regarding ionophore-induced platelet microparticle release, reinforcing the interpretation of our data in the context of research on microparticle release by activated platelets (36). The use of A23187 aids in technique development, producing abundant platelet-derived microparticles (PMP) for clearer observation. Moreover, our efforts extended beyond PMP detection, we have also adapted the methodology to study real-time platelet PS exposure and PMP formation. With this approach, functional studies across various realms, including diseases, functional toxicology, and other aspects related to the physiology and conservation of these species, can be pursued. This opens avenues to further investigate the involvement of these microparticles in different pathologies experienced by animals in their natural habitats or in aquarium settings, aiming to enhance their health, well-being and conservation. In fact, in this work we have demonstrated the usefulness of this assay in research studies, beyond its clinical application. The technique was applied to the evaluation of the effects of aspirin on PS exposure and PMP formation during platelet activation. Specifically, the effect of aspirin on PMP formation has been poorly studied previously. There are previous studies in which the inhibitory effect of aspirin on this process in humans has been documented, however in other studies no effects have been observed (42). Since aspirin blocks the binding of arachidonic acid to COX-1, inhibit its transformation into endoperoxides, which are subsequently transformed into prostaglandins and thromboxane (42). Thromboxane A2 promotes the release of calcium into the cytosol of platelets, with consequent change in shape, platelet aggregation, secretion of granules and release of PMP. So PMP formation is inhibited by aspirin.

As can be seen, the application of this assay can not only help us to diagnose and monitor pathologies in marine mammals but also opens a new field of study around the physiology of the PS exposure and PMP formation process and its alterations induced by contaminants, stress, anthropogenic threats, or diseases.

## 5 Conclusion

Our study on platelet functionality in marine mammals, represents a significant advancement in analytical techniques for this taxonomic group. The development and application for the first time of this novel diagnostic method, exploring PMP levels, phosphatidylserine exposure, and PMP formation in marine mammals, provide a valuable tool for diagnosing platelet disorders in these species. Our study introduces a non-invasive and gentle handling method, preserving optimal platelet function during analysis. This innovative investigation not only enhances diagnostic capabilities but also opens avenues for diverse research applications, benefiting both wild and aquarium-based marine mammals. The adaptation of these assays offers a versatile tool for various studies related to marine mammal health and well-being, addressing limitations in clinical capacities for this taxonomic group. Overall, our work lays the foundation for new diagnostic and therapeutic possibilities in marine mammal health, where clinical capabilities are often constrained.

## Data availability statement

The original contributions presented in the study are included in the article/supplementary material, further inquiries can be directed to the corresponding authors.

## Ethics statement

The animal study was approved by Animal Care and Welfare Committee of the Oceanogràfic. The study was conducted in accordance with the local legislation and institutional requirements.

## Author contributions

MF-B: Writing – original draft, Writing – review & editing, Data curation, Formal analysis, Investigation, Methodology. MV: Writing – review & editing, Data curation. MM: Writing – review & editing, Conceptualization. BJ: Writing – review & editing, Methodology. DG-P: Conceptualization, Writing – review & editing, Data curation, Resources, Writing – original draft. CR-G: Writing – review & editing, Funding acquisition, Supervision, Validation, Writing – original draft. AM-R: Methodology, Writing – review & editing, Conceptualization, Formal analysis, Supervision, Validation. J-EO’C: Conceptualization, Writing – original draft, Writing – review & editing, Investigation, Methodology, Supervision.
